# Dr. Daniel Lewis Taylor

**Published:** 1914-12

**Authors:** 


**Dr. Daniel Lewis Taylor,** Albany 1892, a practitioner of New York City, died at Gilbertsville, July 11, aged 46.

